# Clinical Reasoning in the Absence of a Protocol: A Case Series of Physical Therapy Following Orthobiologic Interventions for Chronic Musculoskeletal Conditions

**DOI:** 10.7759/cureus.115094

**Published:** 2026-08-24

**Authors:** Margaret Klausing, Daniel P McGurren, Hannah Haid, Rita Ator, Celeste Delap

**Affiliations:** 1 Department of Physical Therapy, Nova Southeastern University Dr. Kiran C. Patel College of Osteopathic Medicine, Fort Lauderdale, USA; 2 Department of Physical Therapy, Midwestern University, Glendale, USA

**Keywords:** bone marrow injection, evidence-based management, orthopedics and sports physical therapy, platelet-rich plasma (prp), reflective practice

## Abstract

Physical therapy following orthobiologic interventions occurs in a guidance vacuum, with limited standardized rehabilitation protocols to direct clinical decision-making; in this context, clinical reasoning becomes the operative mechanism through which therapists navigate the uncertainty inherent in postprocedural care. This retrospective case series documents and describes the clinical reasoning processes employed by a physical therapist managing three patients with chronic musculoskeletal conditions following orthobiologic interventions, without evaluating the efficacy of the orthobiologic or rehabilitative interventions. The three cases include 1) a 54-year-old woman with chronic shoulder pain involving a superior labral anterior-posterior tear, rotator cuff tendinosis, and adhesive capsulitis treated with three platelet-rich plasma injections and intermittent physical therapy over eight months; 2) a 38-year-old woman with a 20-year history of knee pain treated with bone marrow aspirate concentrate following arthroscopy and twice-weekly physical therapy for 12 weeks; and 3) a 47-year-old woman with sesamoid avascular necrosis of sesamoid bones of the great toe treated with a sequential series of autologous orthobiologic agents and four physical therapy visits over six months. Cases 1 and 3 demonstrated clinically meaningful improvements exceeding established minimal clinically important difference thresholds on standardized outcome measures and achieved return to prior functional levels, whereas Case 2 demonstrated meaningful functional gains during physical therapy but proceeded to total knee arthroplasty within three months of discharge, illustrating the boundaries of conservative management in advanced joint pathology. Common reasoning patterns across cases included reliance on tissue-response-based progression rather than time-based protocols, individualized loading strategies calibrated to patient-specific factors, and integration of orthobiologic context without allowing theoretical healing timelines to override clinical assessment. These cases illustrate the need for systematic documentation of clinical reasoning to build the practice-based evidence from which future protocols can be derived.

## Introduction

Orthobiologic interventions, including platelet-rich plasma (PRP), bone marrow aspirate concentrate (BMAC), and alpha-2-macroglobulin (A2M), represent a growing category of minimally invasive treatments for chronic musculoskeletal conditions [1,2]. These autologous, biologically derived therapies aim to modulate the local tissue environment, promote healing, and reduce pain through mechanisms that remain incompletely understood [3]. Despite their off-label status for many applications in the United States and variability in regulatory oversight across jurisdictions, clinical uptake has accelerated substantially over the past decade, driven by patient demand and expanding provider interest [1,4]. Physical therapists are increasingly involved in the post-procedural care of patients who have received these interventions, yet the role of rehabilitation within orthobiologic treatment remains poorly defined.

Post-procedural rehabilitation guidance following orthobiologic injections is sparse, inconsistent, and largely absent from the peer-reviewed literature. A recent scoping review by Kruse et al. found that 37.5% of studies examining orthobiologic interventions for osteoarthritis made no mention of any post-procedural protocol, and of those that did, only 38.4% described a rehabilitation component [5]. Formal physical therapy was included in approximately one quarter of described rehabilitation programs, with significant heterogeneity in loading parameters, activity modification timelines, and visit frequency [5,6]. Honbo et al. have outlined tissue-specific loading principles following biologic therapy, emphasizing that early protection and progressive mechanical stimulation are critical to successful outcomes, yet acknowledged that these recommendations remain largely consensus-based rather than evidence-derived [6]. The result is a clinical environment in which physical therapists must make complex decisions about tissue loading, progression, and discharge without standardized protocols to guide them.

When protocols do not exist, clinical reasoning becomes the protocol. Clinical reasoning in physical therapy has been conceptualized as an iterative, adaptive, and collaborative process that integrates cognitive, psychomotor, and affective skills within a biopsychosocial framework [7]. Expert physical therapists employ flexible, context-dependent reasoning strategies, moving fluidly among diagnostic, narrative, collaborative, and procedural reasoning in response to the evolving clinical picture [8], with patient-centered approaches and collaborative reasoning directly linked to superior clinical outcomes [9]. In emerging clinical areas where high-quality evidence is absent, practice-based evidence represents a legitimate and important mode of knowledge generation [10].

The purpose of this retrospective case series is to document the clinical reasoning processes employed by a physical therapist managing patients following orthobiologic interventions for chronic musculoskeletal conditions. The aim is to make implicit decision-making explicit and to inform the development of future rehabilitation frameworks for this emerging clinical area. Functional outcomes are reported descriptively as contextual data; they are not presented as evidence of biologic or rehabilitative efficacy. Because this design includes no comparator group and every patient received both an orthobiologic injection and physical therapy, no causal inference can be drawn about the independent effect of either intervention.

A retrospective case series design was selected to document practice-based reasoning in an emerging clinical area where controlled trials may not be optimal for capturing individualized reasoning processes. The case series is recognized by the Joanna Briggs Institute as a valid form of evidence, particularly when the intent is to describe clinical phenomena or characterize decision-making processes in areas with limited prior investigation [11]. Variability across cases in anatomical site, orthobiologic agent, rehabilitation dosing, and patient self-management capacity was treated as meaningful data rather than a confound, reflecting the individualized nature of clinical reasoning in the absence of standardized protocols.

Cases were drawn from the clinical caseload of a single outpatient physical therapy practice between April 2022 and December 2024. Inclusion criteria were 1) receipt of one or more orthobiologic injections administered by an external provider, 2) completion of a course of physical therapy, and 3) availability of standardized outcome measures at both intake and discharge. Three patients met all inclusion criteria; additional cases were excluded due to incomplete documentation or missing outcome measures. All information was de-identified, and per institutional policy, this activity does not constitute human subjects research; therefore, institutional review board approval was not required. The treating physical therapist (M.K.), a board-certified orthopedic clinical specialist with 10 years of clinical experience in an outpatient orthopedic setting, served as both clinician and author.

Clinical reasoning content was authored directly by M.K. using a structured written reflection format that constitutes a post hoc reconstruction of clinical reasoning rather than a real-time record, addressing initial examination reasoning, impairment prioritization, key decision points, and progression rationale, and was integrated with contemporaneous chart documentation to produce the case-level reasoning narratives presented in the following. Because this reasoning was authored by the treating clinician and reconstructed after the episodes of care, it is subject to confirmation bias, recall bias, and selective reporting; this is the primary methodological limitation of the present series and should be weighed when interpreting the reasoning narratives below.

Outcomes were assessed using the Shoulder Pain and Disability Index (SPADI; minimal clinically important difference (MCID): 8-13 points) [12], the Lower Extremity Functional Scale (LEFS; MCID: 9 points) [13,14], the Patient-Specific Functional Scale (PSFS; MCID: 2-3 points) [15,16], the Foot and Ankle Ability Measure Activities of Daily Living (FAAM ADL) subscale (MCID: 8 percentage points) [17,18], and the Numeric Pain Rating Scale (NPRS; MCID: 2 points) [19]. Goniometric range of motion and handheld dynamometry were used descriptively to characterize patient status and monitor progression. Functional outcome improvements exceeded MCID thresholds, although causality cannot be inferred.

## Case presentation

Case 1: shoulder (chronic rotator cuff tendinosis and adhesive capsulitis)

Clinical Presentation

A 54-year-old woman presented with chronic right shoulder pain in the context of a superior labral anterior-posterior (SLAP) tear, supraspinatus and subscapularis tendinosis, and adhesive capsulitis. Her subjective history revealed a delayed and fragmented path to physical therapy, and she demonstrated a strong focus on imaging findings, which shaped early communication strategies. She had received one leukocyte-rich PRP injection before initiating physical therapy, with a second administered mid-course (mid-May of 2023) requiring temporary modification of loading parameters; physical therapy was not involved in injection decisions or timing. At evaluation, she reported pain levels of 0.5-2/10 and demonstrated moderate disability on the SPADI (22%), limited active and passive shoulder range of motion, weakness with pain during rotator cuff testing, asymmetries in glenohumeral joint mobility, compensatory superior humeral head glide during elevation, and insufficient scapular upward rotation.

Clinical Reasoning

The initial examination shaped a rehabilitation hypothesis centered on chronic movement dysfunction and mobility deficits rather than structural pathology alone. The presence of multi-structural pathology shifted focus away from treating any single structure in isolation and toward addressing the dominant clinical presentation through a movement-system lens. The patient's low pain levels (0.5-2/10) suggested she was beyond the highly irritable phase, supporting a progressive, mobility-focused approach. Mobility loss was hypothesized to be the primary driver, with the rationale that restoring glenohumeral motion would reduce guarding, improve joint mechanics, and create the foundation for effective strengthening. Advanced strengthening and higher level activity tolerance were deprioritized until adequate mobility was restored. The presence of multiple structural imaging findings reinforced the need for patient education, framing these findings within the context of modifiable impairments to reduce fear-avoidance behavior.

Key decision point 1 (continuing care despite sporadic attendance): The patient's attendance was sporadic, presenting a clinical dilemma regarding continuation versus discharge. The therapist chose to continue care because the patient demonstrated meaningful benefit when attending, reported positive home exercise program carryover, and consistently described improvements following sessions. Her presentation did not suggest plateau or regression, but rather slower progress consistent with her attendance pattern. Preserving therapeutic rapport was important given her prior care experiences and initial hesitance toward physical therapy. During absences, the therapist reached out to assess intent, and the patient consistently expressed a desire to continue. At discharge, she demonstrated full restoration of range of motion, resolution of pain, and return to her prior level of function.

Key decision point 2 (early transition to progressive loading): Despite the adhesive capsulitis diagnosis, the therapist transitioned relatively early from a primarily mobility-focused approach to progressive strengthening and functional loading. Low pain levels, improving irritability, and tolerance to movement indicated readiness for increased demand; relying solely on a mobility-based approach risked underdosing rehabilitation and delaying return to function. Progressive loading was introduced in a graded manner, including closed-chain and task-specific activities.

Care Progression

Over eight months, treatment evolved from mobility restoration and symptom modulation to a progressively functional program. The mid-May PRP injection required temporary loading modification during anticipated healing stages while promoting movement to prevent further stiffness. As capacity improved, yoga-specific training was incorporated, including upper extremity weight-bearing, closed-chain stability, and overhead control to mirror practice demands. Periods of slower progress were managed through exercise dose adjustments and reinforcement of the home program.

Outcomes

The SPADI improved from 22% to 0% (change of 22 percentage points; MCID: 8-13 points). NPRS decreased from 0.5-2/10 to 0/10. The PSFS improved from 3-5/10 to 9-10/10 (MCID: 2-3 points). Handheld dynamometry demonstrated near-normalized strength. The patient returned to teaching yoga and all prior activities without limitation. These changes exceeded the MCID for each measure; however, in this uncontrolled design, they are reported as descriptive clinical context and cannot be attributed to the physical therapy or the orthobiologic intervention.

Case 2: knee (postarthroscopic BMAC for chronic knee pain)

Clinical Presentation

A 38-year-old woman presented approximately one week postoperatively following knee arthroscopy with BMAC harvested from the iliac crest, in the context of a 20-year history of bilateral knee pain. No repeat biologic injection was administered during the physical therapy course. Her physician had previously recommended total knee arthroplasty (TKA) and anticipated she would likely require it within a relatively short time frame; her decision to pursue BMAC reflected a preference to attempt conservative management first. At evaluation, she demonstrated manageable pain levels, moderate functional limitation, an Örebro Musculoskeletal Pain Screening Questionnaire score of 33 [20], and an LEFS score of 25/80. PSFS scores were 3/10 for walking and 2/10 for biking. The Örebro and WOMAC were administered at initial evaluation as screening and characterization tools; the LEFS and PSFS served as the primary longitudinal outcome measures. Objectively, she presented with limited range of motion (flexion to 98°), quadriceps inhibition, altered gait mechanics, reduced eccentric control, valgus tendencies, and compensatory movement strategies.

Clinical Reasoning

The rehabilitation hypothesis integrated both the International Classification of Functioning, Disability and Health framework and a movement-system diagnostic approach [21]. The patient presented with impairments in joint mobility, muscle performance, and neuromuscular control, leading to activity limitations in walking, stair negotiation, and higher-level functional tasks. From a movement-system standpoint, her presentation was consistent with a tibiofemoral hypomobility and movement coordination impairment syndrome. The BMAC reinforced the need to balance protection of the biological environment with appropriate mechanical loading, and the therapist anticipated that progression might not follow a standard post-arthroscopy timeline.

Key decision point 1 (blood flow restriction training at week 2): At Week 2, pain and irritability had improved sufficiently to allow progression, but significant quadriceps inhibition and limited weight-bearing tolerance persisted. Blood flow restriction (BFR) training was introduced to bridge the gap between early postoperative protection and muscle capacity restoration, providing a meaningful strengthening stimulus at lower loads through a mechanism proposed to facilitate type II muscle fiber recruitment. Continuing with traditional low-load exercises alone risked underdosing the stimulus needed to address quadriceps inhibition; BFR allowed targeted strengthening within current tolerance while respecting surgical and biological healing constraints.

Key decision point 2 (managing an overtraining episode): A load-capacity mismatch was identified when the patient, following formal discharge from physical therapy, reported an acute flare-up of pain in her knee. Specifically, the patient communicated she experienced symptom exacerbation following an intense biking episode that was accompanied by increases in pain and swelling and moderate regression in functional task tolerance. These changes were accompanied by significant range of motion loss and signs of acute injury (swelling), suggesting a genuine setback. The therapist offered to resume treatment with the patient to restore her to a previous level of function, but at this stage, the patient felt inclined to return to her orthopedic physician and ultimately decided to pursue TKA.

Key decision point 3 (recognizing the trajectory toward arthroplasty): Following discharge, the patient informed the physical therapist of the episode, prompting recognition that rehabilitation alone may be insufficient, as underlying joint pathology and symptom recurrence continued to limit higher-level activity. In retrospect, the therapist's reflections shifted from a purely restorative perspective to one emphasizing optimization and preparation, maximizing strength, movement quality, and independence to support long-term function and potentially improve readiness for future TKA. This shift represents a subjective, post hoc reflection rather than an analytic finding. The eventual progression to TKA can be framed as part of the natural history of her condition rather than a failure of rehabilitation; the care provided contributed to meaningful functional improvements and likely positioned her more favorably for surgical outcomes.

Care Progression

Over 12 weeks, rehabilitation progressed from postoperative recovery (range of motion, swelling, quadriceps inhibition, and gait normalization) through targeted strengthening with BFR to progressive loading, neuromuscular control, and correction of movement impairments, including valgus collapse. Discharge timing was determined through shared decision-making once the patient felt she had achieved an acceptable level of function and was confident in managing her rehabilitation independently moving forward.

Outcomes

Within her time in formal physical therapy, the LEFS improved from 25/80 to 65/80 (change of 40 points; MCID: 9 points). The PSFS improved from 2-3/10 to 9/10 (MCID: 2-3 points). Knee flexion improved from 98° to 142°, with improvements in quadriceps and gluteal strength. These within-treatment changes exceeded the MCID for each measure but are reported as descriptive clinical context in an uncontrolled design and cannot be attributed to a specific intervention. Despite these gains, the patient proceeded to TKA within three months of discharge. This event occurred outside the formal physical therapy episode and is reported as follow-up context rather than as a study outcome; no causal relationship to the rehabilitation course can be inferred.

Case 3: foot/great toe (sesamoid avascular necrosis with sequential orthobiologic agents)

Clinical Presentation

A 47-year-old woman presented with chronic left great toe pain associated with sesamoid avascular necrosis (AVN), with symptoms dating back several years. She underwent a structured series of autologous orthobiologic injections over a 12-week period administered by an external provider: plasma therapy at Week 1, A2M at Week 3, BMAC at Week 7, PRP at Week 9, and plasma therapy at Week 12, with Class IV laser therapy as an adjunct before each injection. Physical therapy was initiated concurrently with the injection series. Functionally, she was limited in running, lunging, and prolonged walking, with pain up to 7/10 with activity. PSFS scores at intake were running 3/10, lunging 3/10, walking in non-supportive footwear 0/10, and hiking 0/10. The FAAM ADL subscale score was 70%. At evaluation, she demonstrated decreased loading through the left great toe during gait with compensatory supination and reduced push-off, mobility restrictions at the first metatarsophalangeal (MTP) joint, decreased ankle dorsiflexion, impairments in intrinsic foot strength, and proximal deficits, including hip weakness and altered lower extremity alignment during squatting and lunging.

Clinical Reasoning

From a movement-system standpoint, the patient's presentation was consistent with a first MTP mobility deficit syndrome leading to altered loading strategies and compensatory patterns throughout the kinetic chain. The sesamoid AVN indicated reduced capacity for load tolerance, requiring careful dosing to avoid symptom exacerbation, but the patient's activity level made strict unloading both impractical and unlikely to produce meaningful change. The regenerative injections, while not guaranteed to reverse the pathology, were intended to support tissue healing and, when paired with appropriately dosed loading, were intended to support functional improvement beyond prior interventions. The plan of care emphasized balancing protection and progression, restoring MTP mobility, improving intrinsic and proximal strength, and gradually reintroducing functional loading.

Key Decision Point 1: Prioritizing Proximal Stabilization. The therapist hypothesized that impaired proximal stability was contributing to altered load distribution and excessive loading at the first MTP joint. Assessment findings supported the following: gluteus medius was 4/5 on the left and 5/5 on the right, and gluteus maximus was 4/5 on the left and 4+/5 on the right, with low back discomfort and hamstring cramping suggesting over-reliance on hamstrings relative to gluteals. Gait analysis revealed decreased push-off with reduced terminal stance mechanics and lateral weight shift; during squatting and lunging, dynamic valgus and reduced proximal stability were observed. Proximal strengthening was incorporated as part of a comprehensive strategy alongside concurrent local interventions. During post-injection periods when the toe was relatively unloaded, proximal strengthening could be emphasized without overloading the involved joint.

Key Decision Point 2: Self-Directed Care Model. A lower-frequency model of four visits over six months was adopted based on the patient's demonstrated capacity for independent management, including strong understanding of her condition, prior rehabilitation experience, and high intrinsic motivation. Each visit served as a checkpoint for reassessment and program progression. Education was central, ensuring the patient understood not only what to do but also how to adjust her program based on symptom response. Between visits, monitoring relied on patient-reported outcomes and clear guidance on acceptable symptom thresholds.

Key Decision Point 3: Graded Return to Impact Activities. Return to impact was guided by demonstrated capacity rather than a fixed timeline. The therapist used a general tissue-healing-based staging approach for tendon loading as a staging guide while individualizing decisions. Progression criteria included minimal pain with current activities, absence of symptom escalation, adequate joint mobility, and improvements in strength and neuromuscular control. Impact activities were introduced in a graded manner from lower-level dynamic loading to running, with ongoing patient education on load management and symptom monitoring.

Outcomes

PSFS scores improved from 0-3/10 to 6-10/10: running 3 to 8/10, lunging 3 to 6-7/10, walking 0 to 8/10, and hiking 0 to 10/10 (MCID: 2-3 points). The FAAM ADL subscale improved from 70% to 94% (change of 24 percentage points; MCID: 8 points). Lower extremity and hip strength normalized to 5/5, and great toe mobility improved. The patient completed a 5K run pain-free and returned to hiking, yoga, and recreational activities without limitation. As in the other cases, these changes exceeded the MCID for each measure but are reported as descriptive clinical context in an uncontrolled design and cannot be attributed to the physical therapy or the orthobiologic series. A summary of patient cases, orthobiologic interventions, rehab considerations, and outcomes can be found in Table 1.

**Table 1:** Patient characteristics, interventions, outcomes, and key clinical reasoning decision points A2M, alpha-2-macroglobulin; AROM, active range of motion; AVN, avascular necrosis; BFR, blood flow restriction; BMAC, bone marrow aspirate concentrate; DC, discharge; FAAM ADL, Foot and Ankle Ability Measure Activities of Daily Living; GH, glenohumeral; HEP, home exercise program; HHD, handheld dynamometry; LEFS, Lower Extremity Functional Scale; MTP, metatarsophalangeal; PRP, platelet-rich plasma; PROM, passive range of motion; PSFS, Patient-Specific Functional Scale; PT, physical therapy; ROM, range of motion; RTN, return; SLAP, superior labral anterior-posterior; SPADI, Shoulder Pain and Disability Index; TKA, total knee arthroplasty

Case	Patient characteristics	Orthobiologic intervention	Rehabilitation dosing and key PT interventions	Key functional measures	Outcomes	Key clinical reasoning decision points
Case 1: shoulder	54-year-old woman; chronic right shoulder pain; SLAP tear, supraspinatus/subscapularis tendinosis, adhesive capsulitis; pain: 0.5-2/10 at evaluation	PRP ×3:#1: Before PT; #2: mid-May (mid-course); #3: late course	Intermittent PT q4-8 weeks ×8 months; GH mobilization, soft tissue; progressive shoulder loading; Yoga-specific functional training	SPADI, PSFS, ROM, HHD, strength	SPADI: 22% → 0%; PSFS: 3-5/10 → 9-10/10; full pain-free AROM/PROM; near-normalized strength; RTN to yoga instruction	1) Continued care despite sporadic attendance: patient demonstrated meaningful benefit when attending and positive HEP carryover; therapeutic rapport preservation prioritized given prior care hesitance.2) Early transition to progressive loading: low irritability and functional tolerance supported earlier than-expected shift from mobility-focused to strengthening/functional program despite adhesive capsulitis diagnosis
Case 2: knee	38-year-old woman; 20-year bilateral knee pain history; postarthroscopy for loose body removal; Örebro: 33	BMAC (iliac crest); single event at surgery; PT started ~1 week postoperatively	2×/week × 12 weeks; patellar/tibial mobilization; edema management; BFR strengthening (from Week 2); progressive functional loading	LEFS, PSFS, ROM, strength	LEFS: 25/80 → 65/80; PSFS: 2-3/10 → 9/10; ROM: 98° → 142° flexion; quadriceps/glute strength improved; TKA within 3 months of DC	1) BFR training at Week 2: quadriceps inhibition with insufficient load tolerance for traditional strengthening; BFR bridged the gap between protection phase and muscle capacity restoration. 2) Overtraining episode management: load-capacity mismatch identified after biking episode; intensity/volume temporarily reduced with graded resumption rather than regression. 3) Trajectory toward TKA: reasoning shifted from restorative to optimization/preparatory focus as rehabilitation ceiling became apparent; care reframed as pre-surgical conditioning
Case 3: foot/great toe	47-year-old woman, chronic left great toe pain, sesamoid AVN, pain up to 7/10 with activity	Sequential protocol over 12 weeks: Week 1: autologous conditioned plasma; Week 3: A2M; Week 7: BMAC (iliac crest); Week 9: PRP; Week 12: autologous conditioned plasma; Class IV laser adjunct	4 PT visits over 6 months, self-directed program, first MTP mobilization, intrinsic foot strengthening, proximal glute/core strengthening, graded return to impact	PSFS, FAAM ADL, ROM, strength	PSFS: 0-3/10 → 6-10/10; FAAM: 70% → 94%; Strength normalized 5/5; great toe mobility improved; completed 5K pain-free; RTN hiking, yoga, running	1) Proximal stabilization focus: glute med 4/5 L vs. 5/5 R with dynamic valgus and decreased push-off during gait; proximal deficits contributing to abnormal distal loading patterns at the first MTP. 2) Self-directed care model: high health literacy, prior rehabilitation experience, and strong intrinsic motivation supported low-frequency visits with self-managed progression between checkpoints. 3) Graded return to impact: Mayo Clinic PRP tendon protocol used as general staging guide; progression criteria based on pain, ROM, strength, and movement quality rather than a fixed timeline

## Discussion

**Figure 1:**
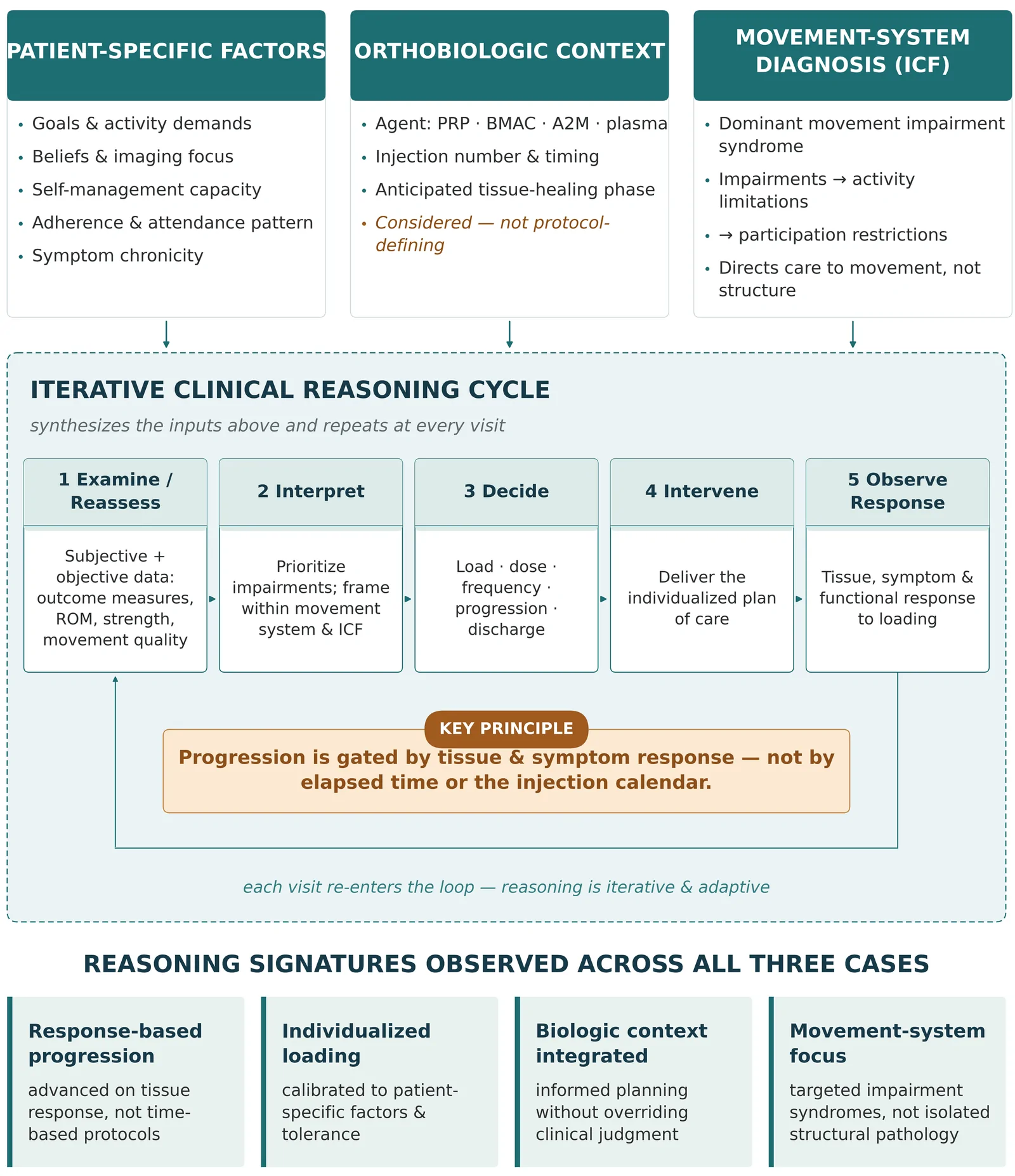
Clinical reasoning as the operative framework in postorthobiologic rehabilitation Conceptual model of the clinical reasoning process applied by the treating physical therapist across all three cases in the absence of standardized postorthobiologic rehabilitation protocols. Three input domains (patient-specific factors, orthobiologic context, and a movement-system diagnosis framed within the ICF) were integrated within an iterative reasoning cycle (examine/reassess → interpret → decide → intervene → observe response) repeated at each visit. The key principle governing the model, highlighted at center, is that progression was advanced according to tissue and symptom response rather than elapsed time or a fixed injection schedule. The bottom panel summarizes the reasoning signatures common to all three cases. The figure depicts the reasoning process only; functional outcomes are reported descriptively as clinical context and are not presented as evidence of orthobiologic or rehabilitative efficacy [7-9] A2M, alpha-2-macroglobulin; BMAC, bone marrow aspirate concentrate; ICF, International Classification of Functioning, Disability and Health; PRP, platelet-rich plasma; ROM, range of motion Image credit: This is an original image created by the author Daniel P. McGurren using Claude (Anthropic, San Francisco, CA)

Synthesis of clinical reasoning documented across the three cases revealed several recurring patterns that characterize how an experienced therapist navigated the absence of standardized post-orthobiologic rehabilitation protocols, consistent with the clinical reasoning literature establishing that expert physical therapists employ flexible, context-dependent reasoning strategies rather than rigid, protocol-driven approaches [7-9]. The clinical reasoning process shared across the three cases is summarized conceptually in Figure 1, which maps the recurring sequence of tissue-response assessment, individualized loading decisions, movement-system diagnosis, and iterative reassessment that guided care in each patient.

Across all three cases, the treating therapist used patient response rather than procedural calendars or injection-specific timelines to guide progression decisions. In Case 1, the mid-course PRP injection required temporary modification of loading, but progression resumed based on clinical response rather than a fixed post-injection timeline. In Case 2, loading decisions following the surgical BMAC procedure were guided by symptoms and loading tolerance as well as quadriceps activation. In Case 3, the sequential injection protocol over 12 weeks required ongoing calibration of loading around each injection, with tissue tolerance as the primary driver of advancement.

Rehabilitation contact patterns varied markedly, ranging from twice-weekly sessions over 12 weeks (Case 2) to four visits over six months (Case 3). Case 1 unfolded over eight months of intermittent attendance, which the therapist chose to accommodate rather than respond to with discharge, a reasoning decision weighing therapeutic rapport, home-program carryover, and incremental progress against traditional dose parameters. These differences reflected integration of tissue tolerance, patient goals, self-management capacity, and pathological context into individualized care-delivery decisions.

Across cases, the therapist considered proposed biologic mechanisms in rehabilitation planning but did not allow theoretical tissue healing timelines to override direct clinical assessment. The therapist maintained awareness of the biological environment, avoiding excessive loading during anticipated inflammatory and proliferative stages, while using actual clinical response as the decisive factor for progression. Additionally, all three cases employed a movement-system diagnostic approach, directing interventions toward dominant movement impairment syndromes rather than isolated structural pathology, glenohumeral mobility restoration over structure-specific SLAP treatment in Case 1, tibiofemoral movement coordination impairments in Case 2, and first MTP mobility deficit syndrome with proximal contributions in Case 3.

Case 2 warrants particular discussion. Despite LEFS improvement exceeding the MCID by a factor of 4, the patient proceeded to TKA within three months of discharge. This outcome illustrates the boundaries of what rehabilitation reasoning can achieve in advanced structural joint degeneration with a 20-year symptom history. The trajectory toward surgery does not permit any claim about whether that reasoning was optimal; it does, however, highlight important considerations about patient selection for orthobiologic intervention, the role of shared decision-making in setting realistic expectations, and the limits of tissue-level healing in advanced joint pathology. Reporting this outcome transparently strengthens the clinical and scholarly integrity of this case series.

An incidental observation is that all three patients were female and peri- or postmenopausal at the time of treatment. Hormonal changes associated with menopause are known to influence musculoskeletal tissue properties, and emerging evidence suggests these factors may affect tissue healing capacity and response to both orthobiologic and rehabilitative interventions [22]. Although no conclusions can be drawn from this small case series, this observation underscores the need for future research examining how sex- and age-related variables may influence recovery trajectories following orthobiologic procedures.

These cases carry two distinct implications. For clinical practice, making clinical reasoning explicit through structured documentation is itself a form of knowledge generation in areas where formal evidence is absent. For research, prospective studies should capture not only functional outcomes but also the reasoning processes that drive intervention decisions; think-aloud and stimulated recall methodologies offer a promising approach for studying how therapists navigate post-orthobiologic rehabilitation in real time [8].

Several limitations should be interpreted in the context of this study’s purpose, which is to document clinical reasoning rather than to establish efficacy or generalizability. First, the sample is small (n = 3) and demographically homogeneous (all female, peri/postmenopausal), limiting the transferability of findings to other populations. Second, clinical reasoning was reconstructed retrospectively through the treating therapist’s own structured written reflection, authored as a co-author of this work. This approach captures the therapist’s reasoning as she reconstructs it post hoc and may not fully represent real-time cognitive processes; prospective designs using think-aloud or stimulated-recall methodology would better capture in-the-moment reasoning. Third, all orthobiologic interventions were administered by external providers, and the treating therapist had no control over injection quality, timing, agent selection, or procedural protocol. Fourth, the natural history of each condition cannot be ruled out as a contributor to observed improvements. Fifth, inclusion criteria required complete documentation, which may introduce selection bias toward patients with more thorough chart records.

## Conclusions

In the absence of standardized post-orthobiologic rehabilitation protocols, clinical reasoning served as the primary decisional framework, with the treating therapist relying on tissue response assessment, individualized loading strategies, movement impairment system-based diagnosis, and adaptive decision-making to guide care across three distinct clinical presentations. These cases underscore the need for systematic documentation of clinical reasoning in emerging clinical domains and highlight the value of making implicit decision-making explicit through structured reflection. Physical therapists working in post-orthobiologic rehabilitation may consider documenting their reasoning systematically, which these cases suggest can contribute to the evidence base from which future rehabilitation guidelines may be developed. The observations reported here are intended to generate hypotheses and inform future research rather than to establish best practice.
